# Association between socioeconomic status of mothers, food security, food safety practices and the double burden of malnutrition in the Lalitpur district, Nepal

**DOI:** 10.1186/s13690-016-0150-z

**Published:** 2016-09-13

**Authors:** Mahesh Sarki, Aileen Robertson, Alexandr Parlesak

**Affiliations:** 1 Global Nutrition and Health, Metropolitan University College, Pustervig 8, 1126 København K, Denmark; 2 Nutrition Promotion and Consultancy Services (NPCS), NGO, Kathmandu, Nepal; 3 WHO Collaborating Centre for Global Nutrition and Health, Copenhagen, Denmark

**Keywords:** Stunting, Childhood overweight, Childhood obesity, Women’s education, Food safety

## Abstract

**Background:**

The prevalence of childhood overweight and obesity is increasing in low-and middle income countries such as Nepal. At the same time, high prevalence of chronic undernutrition persists leading to a double burden of malnutrition.

**Aim:**

To identify associations between the socioeconomic status of mothers, food security, the food safety environment within the household, and prevalence of stunting and overweight of the children.

**Methods:**

Statistical analysis of socioeconomic, food safety-related and anthropometric data from 289 mother-child dyads in an urban area of the Kathmandu Valley, Nepal.

**Results:**

According to WHO standards, 26 % of the children, aged 0–59 months, were stunted, 10 % were underweight, and 6.6 % were either overweight or obese. Significantly more boys than girls were underweight (*p* = 0.004) and stunted (*p* < 0.001). The higher education level of mothers was associated with a higher height-for-age (HAZ) score in girls, but not with HAZ in boys. Irrespective of sex, children of mothers with highest education level had significantly lower BMI-for-age scores (BAZ) than those of mothers with low education levels. None of the food safety indicators were associated with either HAZ or the BAZ.

**Conclusion:**

The education level of mothers seems to be relevant to help reduce the double burden of malnutrition at least in some regions of Nepal. This should be taken into consideration when designing programmes to prevent both chronic undernutrition and non-communicable diseases.

## Background

Levels of stunting in young children are declining however where levels are still high a double health burden is developing in low and middle-income countries (LMICs) such as Nepal. Recent estimates show that globally about 13 million infants are born with intra-uterine growth retardation, 112 million children below the age of 5 years are underweight and 178 million are stunted, a large majority of which live in South East Asia [1]. Undernourished children experience an irreversible reduction in physical and cognitive development, become highly prone to infections and vulnerable to chronic diseases [2].

Diarrhoeal diseases are the leading causes of mortality, morbidity and stunting among young children in LMICs and Nepal is among the World’s top ten countries having the highest prevalence of stunting [1]. The lack of safe water, basic sanitation and hygiene accounts for as much as 88 % of the disease burden from diarrhoea [3] and, particularly in LMICs, diarrhoea increases the risk of becoming wasted [4]. Despite adequate nutrition, wasting and stunting can persist in children from LMICs and lack of good sanitation is believed to be an essential cause [5, 6].

LMICs such as Nepal are undergoing nutritional transition where chronic undernutrition (stunting) is occurring along with emerging prevalence of overweight and obesity, creating a double burden of nutrition-related diseases [7]. Obesity in childhood is linked with increased risk of adult obesity and chronic disorders, including hypertension, cardiovascular disease, glucose intolerance and metabolic syndrome, and increased risk of mortality [8]. Escalating rates of childhood obesity are increasingly adding burden to the health cost of many resource-poor countries such as Nepal [9].

Data on overweight and obesity among children in Nepal are scarce but statistics from nearby countries indicate childhood obesity to be surprisingly high. The prevalence of childhood overweight/obesity in South Asian countries is increasing [10], reaching prevalence estimates as high as 26 % overweight and 14 % obese in Bangladesh [11], 17 % overweight and 7.5 % obese in Pakistan [12], and 19 % overweight and 5.3 % obese in India [13]. Between 36 and 38 % of the South Asian population are either over- or underweight [14–16]. A cross-sectional study carried out in the Lalitpur district of Nepal found 14 % of private school children to be overweight and 11.3 % to be obese [17]. In some LMICs in South-East Asia, mothers’ education was found to be associated with their children’s body-mass index [18].

The aim of this cross-sectional study was to analyse associations between the education level and the employment situation of the mother, the food safety conditions within the household and the prevalence of stunting and overweight among young children.

## Methods

### Study participants

All data were recorded by trained community health volunteers in March 2014 in ward 22 of Lalitpur district, an urban area near Kathmandu, Nepal. This ward was selected because it’s one of the least developed wards and requires particularly high input of health services and welfare benefits from the public health unit of LSMC. There are 22 Wards in LSMC and Ward 22 contains 13 *Toles* (districts). In total, data from 294 mother-child dyads were recorded but 5 were excluded due to missing information. All mothers from 13 *Toles* of Ward 22, who had at least one child younger than 5 years of age, were interviewed for the survey by door-to-door contact. The applied questionnaire was pretested in an urban setting, revised and then pilot-retested. A group of 10 local community health volunteers (*Tole* Health Promoters, THPs, and Ward Health-In-Charges, WHIC) from ward 22 of the Lalitpur district were recruited by the local Public Health Office. These recruits attended an intensive 3 day training on survey methodology, including administration of the questionnaire and how to carry out anthropometric measurements of mothers and their children. The ten trained recruits were divided into groups of a minimum of three, which were accompanied by 3 staff members of the Nutrition Promotion and Consultancy Services (NPCS) to supervise data collection during the entire period, All groups had both male and female enumerators and most of the interviews were conducted in Nepali but a few were conducted in *Newari* language, the language of the majority of people living in that Ward.

The questionnaire contained questions on: self-assessed food security; food safety; supplement intake; health check-ups; infant feeding practices; along with anthropometric measurements of the mother and her child, if under 60 months of age. Demographic information such as the age of the mother at birth of her firstborn, the number of family members, the number of children in the family (segregated by sex), and the time since the birth of her youngest child (3-level scale) were recorded. Antenatal iron supplementation (8-level scale) for the child in question was also documented.

Food security was assessed based on having enough money to cover food expenses (4-level scale), availability of desired food (2-level scale), and land ownership (2-level scale). To avoid bias of using only one question to assess food security, the consistency of answers to these three questions was checked before analysis. The following food safety indicators were recorded: self-reported habit of washing hands in the kitchen and the toilet for the child and the mother, with or without soap (2 × 3 levels); water and soap availability at interview (3 levels); the type of water source (bought/tap/well/other); the method used to decontaminate water (boiling/filter/sunlight-UV/untreated); storage of food (in the fridge, covered or uncovered); and cleaning procedures for fruits before consumption (washing, or not, with water of different safety levels). A possible bias when recording the food safety parameters was that some women could misreport that they washed their hands with soap and water after toilet use. To overcome this bias, data collectors were asked to check toilets or hand washing basins in each household to see if soap was present or not. The data collectors sought permission from the mothers before checking their toilets or hand washing basins.

The socio-economic status was assessed based on the duration of the women’s education, their employment situation, and the ownership of land. The mothers’ education level was grouped into the following categories: illiterate, attending literate classes for adults, elementary school, secondary school, and any education going beyond secondary school. The professional activity of the women was assigned to one of the following categories: housewife (without profession), employee with a rather sedentary lifestyle, running own business (usually maintaining small selling booths), and workers doing physically demanding labour.

Anthropometric indices were recorded using a calibrated balance (Seca 874 U, Hamburg, Germany) and a stadiometer (UNICEF, S0114400 Height measuring instrument). The weight and the height, the z-scores of the height-for age (HAZ), the weight-for-age (WAZ) and the body-mass-for age (BAZ) were calculated using WHO AnthroPlus [19] where the age of the child was calculated as the difference between the date of recording and their date of birth. Moderate or severe stunting of the children were defined according to WHO standards [20]. Accordingly, severe and moderate underweight were classified using the WAZ, while overweight and obesity were defined on the basis of the BAZ [21].

A data collector from each group had to review the completed questionnaires to ensure accuracy of data collection and recording at end of each day.

### Statistics

The dependence of HAZ and BAZ of the children and the BMI of the mothers on the recorded (independent) data was evaluated with multivariable linear regression using a step-wise backward removal procedure (threshold *P* value: 0.05). First, a complete multivariable linear regression model was built including all recorded variables considered to be linked to stunting, overweight, or obesity. Then, in subsequent steps, the variable that was the least significant was recursively removed until the multivariable regression model contained independent variables with significant betas only (= minimum adequate model, MAM). Ordinal variables (e.g. education level, food safety level of drinking water) were included into the regression analysis due to their rank. Two-level categorical predictor variables (e.g. male/female, yes/no) were subjected to sigma-parametrization, which means that they were re-coded to values of 1 or −1 and then implemented into the multivariable linear regression analysis. Correlations with a *P* value below 0.05 were considered as significant. For multi-group comparisons, ANOVA was applied with Fisher’s LSD post-hoc test. Simple correlations (BMI of the mothers vs. HAZ and BAZ of the children) were analysed with Pearson’s test. *P* values of distribution inhomogeneity (crosstabulation testing) were calculated with Fisher’s exact test. Software packages used were STATISTICA® V. 12.1 (StatSoft Inc., Tulsa, OK) and [R] V. 3.1.1 (The R Foundation, Vienna, Austria) along with RStudio V0.98.987 (RStudio Inc., Boston, MA).

## Results

The anthropometric indices of children and their mothers’ body-mass index (BMI) are shown in Table 1. The predominant number of children per family was one (*N* = 256), 25 families had 2 children (*N* = 25) and only 8 families had 3 children (*N* = 8) (Table 1).

**Table 1:** Anthropometric indices of the investigated mother-child dyads (*N* = 289) in the Lalitpur district, Nepal, spring 2014

	Mean	Minimum	Maximum	SD*
Number of children in the family	1.14	1	3	0.42
Age of child (months)	24.3	1	60	15.7
Age- and sex-adjusted Z-score of height (HAZ)	−0.87	−8.59	11.5	1.89
Age- and sex-adjusted Z-score of the body-mass index (BAZ)	0.13	−4.75	5.56	1.35
Mothers’ body-mass index (BMI)	24.4	15.6	35.9	4.23

*SD: standard deviation

Twenty-six percent of the children were severely or moderately stunted. Significantly more boys than girls suffered from moderate stunting and moderate underweight (Table 2). The prevalence of children being either overweight or obese was 6.6 %. Almost two thirds (63.6 %) of the children were not stunted, underweight, overweight, or obese (Table 2). Ten children (3.5 %) were both stunted and overweight.

**Table 2:** Prevalence of stunting, underweight, overweight, and obesity in boys (*N* = 176) and girls (*N* = 113) in the Lalitpur district, Nepal, who participated in the study during spring 2014. *P* values indicate the significance levels from Fisher’s Exact Test

	Boys (n)	Girls (n)	Relative boys (%)	Relative girls (%)	*P* value
Moderately stunted^a^	39	11	22.2	9.7	<0.001
Severely stunted^b^	13	11	7.4	9.7	0.526
Moderately underweight^c^	19	5	10.8	4.4	0.004
Severely underweight^d^	4	2	2.3	1.8	1.000
Overweight^e^	8	5	4.5	4.4	0.950
Obese^f^	2	4	1.1	3.5	0.220

^a^: HAZ < −2 and ≥ −3 standard deviation units (SDs or Z scores) below the median. ^b^: HAZ < −3 SDs below the median. ^c^: WAZ < −2 and ≥ −3 SDs below the median; ^d^: WAZ < −3 SDs below the median; ^e^: BAZ > 2 SD but ≤3 SDs above the median; ^f^: BAZ > 3 SDs above the median

General linear regression modelling suggests that a higher HAZ is associated with the following factors: the number of boys living in the family, the mother’s education level, family’s ownership of land, and whether the investigated child is a girl (Table 3). Stunting (HAZ ≤ 2 Z-scores) is associated with unemployment of the mother and the length of the time since the youngest child in the family was born (Table 3). Female sex of the child was associated with a higher HAZ.

**Table 3:** Significant (*p* < 0.05), standardized correlation factors (betas, ß) of multivariable regression analysis of z-scores of children’s height (HAZ) and body-mass index (BAZ) and social indicators of their mothers living in the Lalitpur district, Nepal (spring 2014) in the minimum adequate model (MAM)

	HAZ			BAZ		
Effect	Beta (ß)	Standard error (ß)	*P* value	Beta (ß)	Standard error (ß)	*P* value
Number of children/family				−0.135	0.062	0.029
Number of boys/family	0.185	0.087	0.035			
Education level of the mother	0.182	0.059	0.002	−0.147	0.062	0.018
Mother is unemployed	−0.148	0.060	0.014			
Mother has a paid (mostly hard labour) job				−0.168	0.061	0.006
The family owns land	0.143	0.060	0.018	−0.167	0.061	0.006
Time passed since last pregnancy	−0.213	0.060	<0.001			
Investigated child is a girl	0.314	0.086	<0.001			

**Fig. 1:**
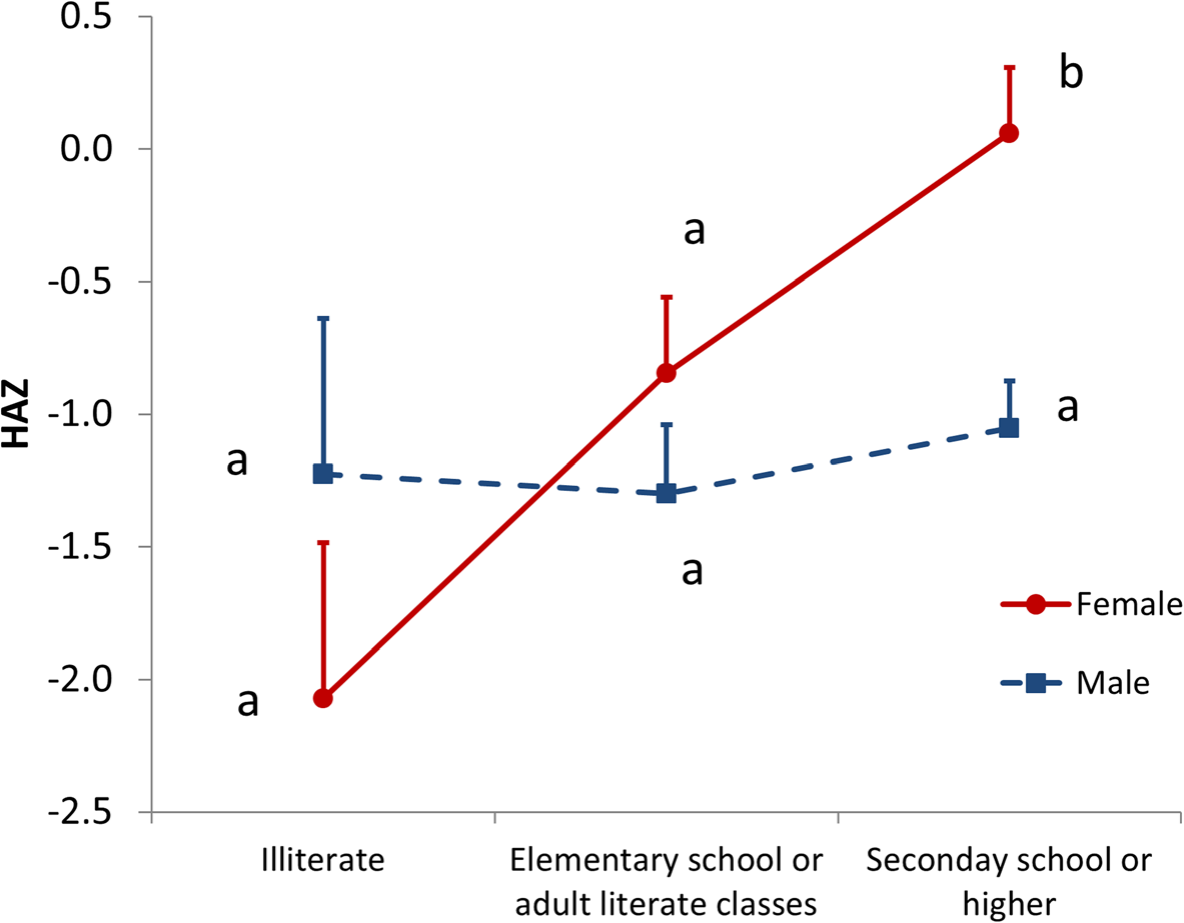
Education status of mothers and HAZ of their children, segregated by sex, in the Lalitpur district, Nepal as recorded in spring 2014. Points indicate the average values plus their standard errors. Different letters assign values that are of statistically significant difference. Significance calculations were done with a 2-way ANOVA and the LSD post-hoc test

The significant positive correlation between the education level of the mothers and their children’s HAZ found in the multivariable regression was confirmed in univariable analysis (Fig. 1). The HAZ of children of illiterate mothers was the lowest (both girls and boys: −1.65 ± 2.22) and the HAZ values for children of mothers having a secondary school education or an education beyond secondary school were the highest (both girls and boys, -0.61 ± 2.24 and −0.76 ± 1.83, respectively). The effect of the mothers’ education on HAZ depends on the sex of the child: while the boys’ HAZ doesn’t differ significantly among the three education categories, the girls’ HAZ scores rise as their mothers’ education level increases (Fig. 1).

**Fig. 2:**
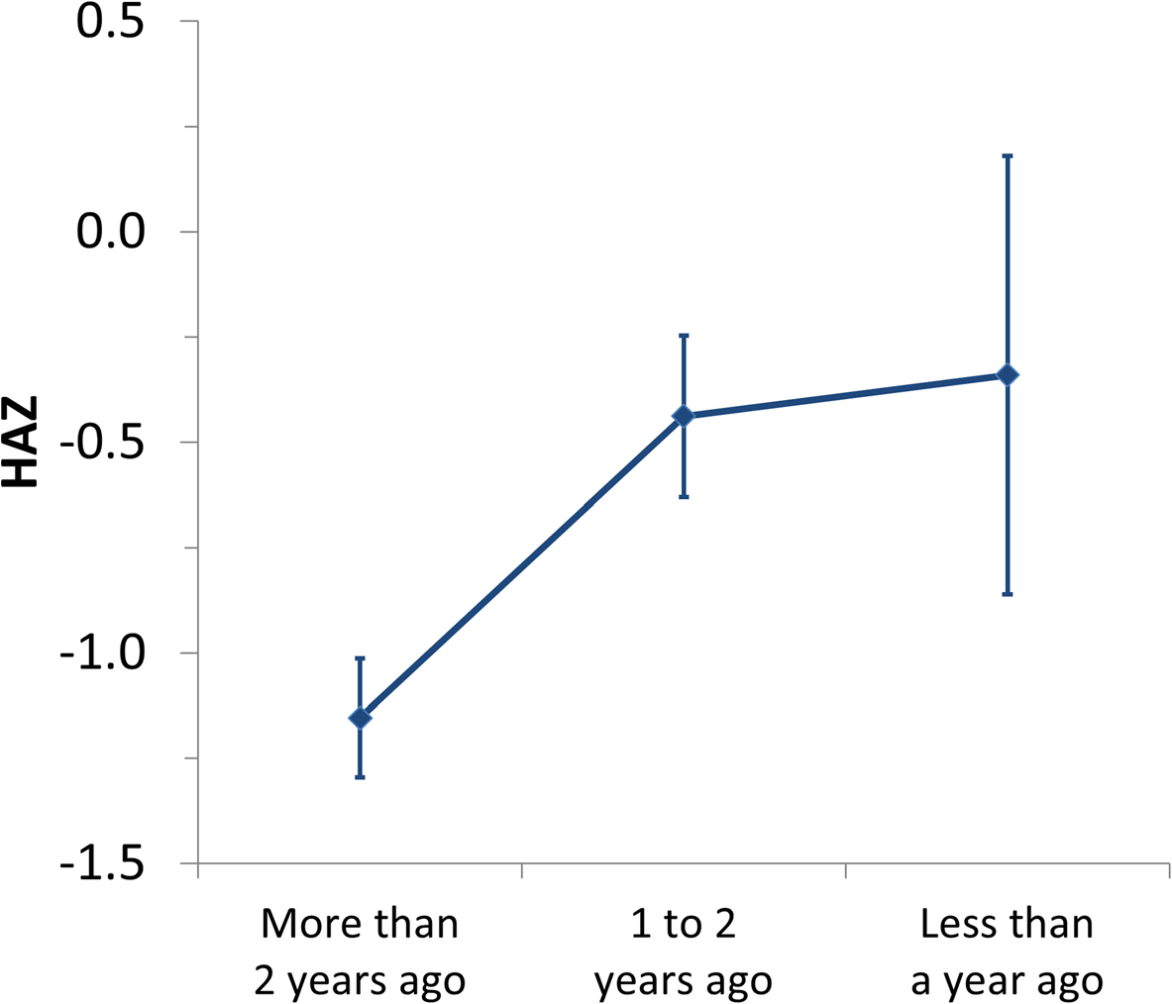
Effect of the time since the last child was born on the children’s HAZ in the Lalitpur district, Nepal, in spring 2014. Values are averages ± standard errors

The longer the period since the last child in the family was born the lower was the HAZ of the children (Fig. 2, *P* value from ANOVA: 0.007). Although indicated as significant in the explorative multiple regression analysis, covariating factors for HAZ, i.e. the number of boys in the family, the fact whether the mother is unemployed or whether the child’s family owns land, did not affect the HAZ significantly when analysed by ANOVA.

None of the indicators associated with hygiene and food safety (water source and purification, availability and usage of soap, refrigeration, hand cleaning habits, washing of fruits) showed a significant correlation with the children’s HAZ.

Exploratory multivariable regression analysis indicated that the sex- and age-standardized body-mass index (BAZ) decreased with: the total number of the children in the family; the education level of the mother; the mother’s unemployment; and land ownership (Table 3).

**Fig. 3:**
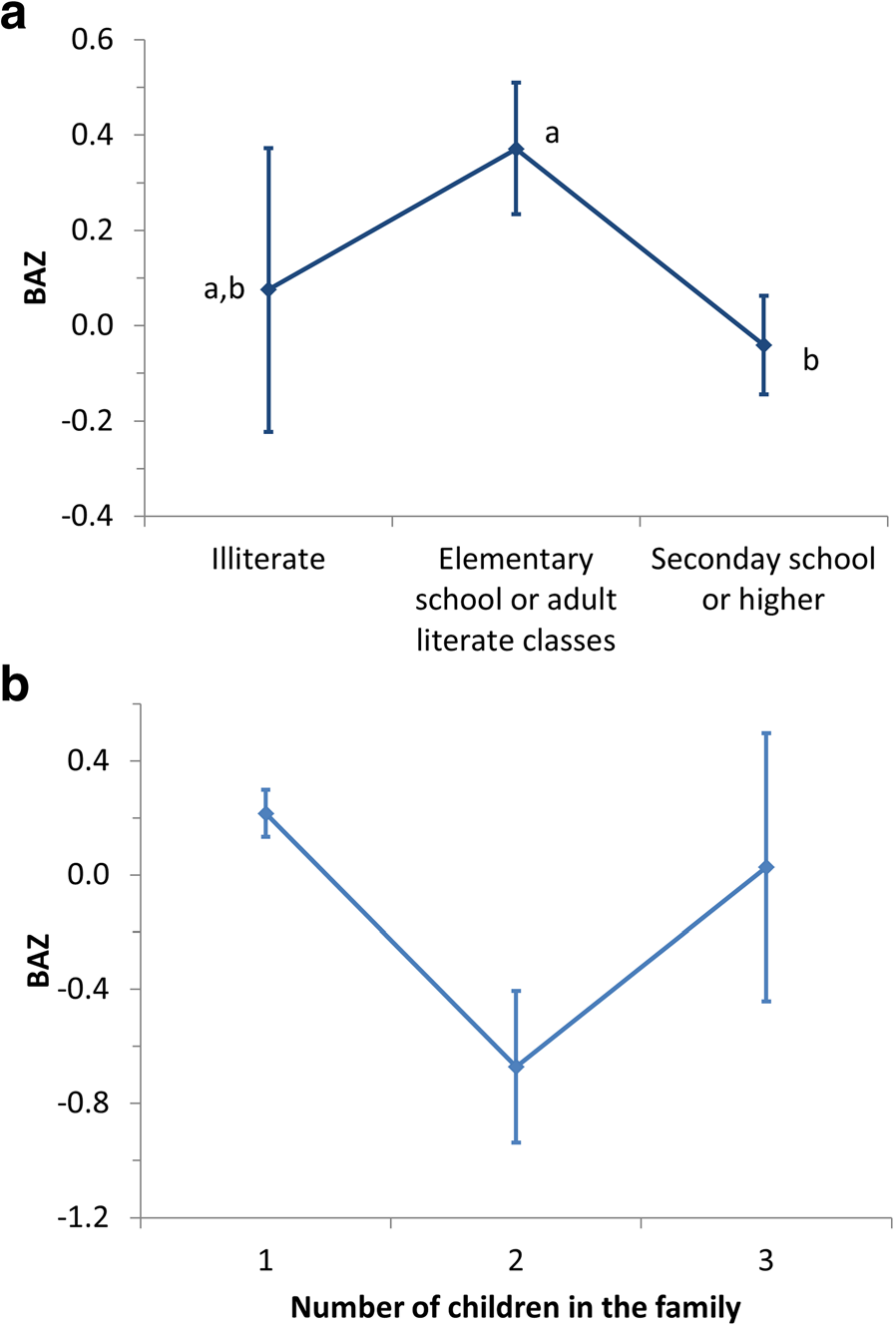
Association of the children’s BAZ with education status of their mothers (**a**) and with the number of children in the family (**b**) in the Lalitpur district, Nepal, in spring 2014. Points labelled with different letters indicate significantly different (*P* < 0.05) values

The BAZ of children with mothers having a secondary school degree, or higher, was significantly lower than that of mothers having only an elementary school education or those who have visited literate classes for adults (Fig. 3a). The BAZ of children living with one sibling was significantly lower than that of single children (*P* = 0.002, Fig. 3b). Children with mothers who had to work hard physically had a significantly (*P* = 0.041) lower BAZ (−0.65 ± 0.08) as compared to children with mothers without strenuous physical activity (0.16 ± 0.33). If the child’s family owns land, the child’s average BAZ (−0.05 ± 0.11) was significantly lower than if this was not the case (0.41 ± 0.11).

As with HAZ, the BAZ was not associated with any of the recorded indicators of food safety.

Thirty per cent of the mothers were overweight (25 kg/m^2^ ≤ BMI < 30 kg/m^2^) and 12 % were obese (BMI ≥ 30 kg/m^2^). If classified according to a WHO expert consultation, which stated for Asian populations potential public health action points to be 23 kg/m^2^ and 27.5 kg/m^2^ [22], 35 % of the investigated mothers were at moderate to high risk to develop the metabolic syndrome (23 kg/m^2^ - 27.49 kg/m^2^) and 23 % had a high to very high risk (≥27.5 kg/m^2^). The mothers’ BMI correlated only moderately with the children’s BAZ (*r* = 0.130, *P* = 0.027) and did not correlate with the HAZ (*r* = 0.005, *P* = 0.845). General linear modelling did not reveal any significant associations between the factors analysed and the mothers’ BMI.

## Discussion

As the group of selected mother-child dyads originated from a *Tole* that is in special need of health services and welfare benefits, no extrapolation of the results can be made for the Nepalese general population. However, the prevalence of the measured risk factors seems comparable to some other parts of Nepal [23]. One of the key findings of this study is that a higher education level of mothers is more closely associated with reduced levels of stunting in their female offspring and not with the hygienic conditions. The absence of an association between hygienic conditions and stunting may indicate that hygiene may not be a key determinant of the levels of chronic undernutrition found in this region. Food safety conditions in Nepal continue to improve. By 2011 only 11 % of households obtained their water from unprotected wells/springs or surface water, and 57 % of households had achieved minimum standard toilet facilities [23]. There has been a considerable improvement and this seems to be confirmed in this study by the absence of an association between poor hygiene and levels of stunting.

Improved education of the mothers seems to be a protective factor against stunting in girls in this study. In previous studies from LMICs, low education of mothers has been linked with their children’s stunting, wasting and underweight [24, 25]. Over recent years, the increased enrolment of Nepalese girls into school has been noteworthy [26]. More females have been involved in the labour market and this has helped Nepal’s Gini index improve from 0.43 to 0.33 between 2000 and 2012 [26]. Similarly Nepal has over the past 15 years made significant progress towards achieving their Millennium Development Goals (MDGs) in maternal and child health [26]. The levels of underweight in children are close to 29 %, the Nepalese MDG 4 goal for 2015 [23]. Similarly the level of stunting decreased by 14 % between 2001 and 2006, and a further 16 % reduction was observed between 2006 and 2011. This is partly explained by the considerable extension of health services such as antenatal care, family planning, and delivery services [26]. This present study suggests that an improvement in the mothers’ education is an additional protective factor against chronic undernutrition and stunting in young girls.

In Pakistan, mothers with virtually no school education parent the majority of malnourished infants, which is partly explained by the introduction of complementary foods at an inappropriate age [27]. However in the present study the association of the mothers’ education with their children’s stunting level depends on the sex of the child: while the boys’ HAZ doesn’t differ significantly among the mothers with different education, the girls’ HAZ rises if their mothers’ education increases. This phenomenon of sex-specific differences in offspring has been observed in countries other than Nepal. In China, where son preference is high, male offspring showed a sex-adjusted height advantage compared to their female counterparts while this advantage does not occur where son preference is low e.g. in the Philippines [28]. Traditionally, Nepali parents prefer to have a baby boy [29] and this may lead to an education-independent favouring of boys in Nepali families. If this is expressed also at the level of nutrition, the HAZ of boys may not differ significantly between mothers of differing education levels as they care and feed all boys similarly. A motivation for the traditional favouritism of boys may be associated with cultural and economic roles in Nepalese society as the son inherits the parental property and has to take care of his elderly parents [29]. Moreover, the majority of people in the Lalitpur district adhere to Hindu religion (~74 %) [30] where the birth of a female child is considered disadvantageous [29]. Thus higher education levels of the mother appear to reduce male gender favouritism. Indeed in this study it appears that the feeding of girls may be even better than of boys, especially in mothers with the highest level of education. These findings may suggest that educated mothers are more aware of gender equality, care, and cultural misconceptions.

In the present study, low unemployment levels of mothers were associated with low stunting levels in their children. This is in accordance with several studies from South Asia and other developing countries, which report that children of employed mothers are less likely to be stunted [31, 32].

In this study mothers who have been to secondary school or have a higher education had children who were significantly less frequently overweight and on average had a lower BAZ, compared to mothers who went to only elementary school or had to visit adult literacy classes. These findings suggest that children of better educated mothers are less likely to be overweight/obese or underweight. Better educated mothers may be more receptive to nutritional education and be able to prevent nutritional related problems. In Mexico and in Ethiopia, a higher education level in mothers seems to reduce the onset of their children’s overweight and obesity [33, 34]. However, there are exceptions to this and in some cultures a higher education of the mother can be associated with higher levels of overweight in their children [35].

In this study the children’s sex-and age-standardized body-mass index (BAZ) decreased along with the number of children in the family, mother’s unemployment, and land ownership. The reduced BAZ of children with parents owning land may be due to the fact that the children may have less access to highly processed and energy-dense foods as compared to those in the city area [36], which may protect them from becoming overweight or obese. Indeed in Uganda, efforts to enhance access to land for urban farming improved the quality of dietary diversity and intake among urban residents [37]. In addition children living in families owning land may be involved in field work, leading to a higher physical activity level, a recognised protective factor against overweight and obesity [9].

The inverse correlation found here between the weight (BAZ) of the children and the number of children in the family has also been observed in a previous study from Nepal [17], where children who had 2 or less siblings were more likely to become overweight or obese compared with children from families that had 4 or more children. Similar results were obtained in a study in Bangladesh [38] and in both studies reduced food security, within families with many children, was explained by reduced share of food per sibling [39].

### Limitations of the study

This study has all the limitations that are valid for cross-sectional studies and further longitudinal and intervention studies are needed to provide final evidence for the assumptions indicated above. However, parallel findings from other LMICs support the idea that a low education level of mothers is a relevant factor for the development of double burden of malnutrition.

## Conclusion

The results of this study indicate that about one third of Nepalese children in the Kathmandu Valley are stunted, underweight, overweight, or obese. Girls who are less stunted seem to profit from their mothers having a higher education level. Moreover, children of both sexes having mothers with the highest education level are significantly less overweight or obese (lower BAZ) compared with the children of mothers with a lower education level. Hence, in order to effectively reduce the double burden of both under- and overnutrition in Nepal, better education of illiterate women and girls attending elementary school seems to be a key factor.

## Abbreviations

ANOVA, Analysis of variance; BAZ, BMI-for-age z score; BMI, Body-mass index; HAZ, Height-for-age z-score; LMIC, Low-to- middle income country; LSMC, Lalitpur Sub-Metropolitan City; MDG, Millenium Development Goal; NPCS, Nutrition Promotion and Consultancy Services; THP, Tole Health Promoter; UNICEF, United Nations Children’s Emergency Fund; WAZ, Weight-for-age z score; WHIC, Ward Health-In-Charges; WHO, World Health Organization; WMA, World Medical Association
